# Testicular necrosis as a complication of severe epididymo-orchitis: a case report

**DOI:** 10.11604/pamj.2022.42.148.35560

**Published:** 2022-06-23

**Authors:** Faisal Ahmed, Fayed Al-Yousofy, Qasem Alyhari, Iman Alyabary, Shams Alzubairi, Saleh Al-Wageeh, Ebrahim Al-Shami, Menawar Dajenah, Ahmed Al-Hitari

**Affiliations:** 1 Department of Urology, School of Medicine, Ibb University of Medical Sciences, Ibb, Yemen,; 2 Department of Pathology, Faculty of Medicine, Taiz University of Medical Science, Taiz, Yemen,; 3 Department of General Surgery, School of Medicine, Ibb University of Medical Science, Ibb, Yemen,; 4 Department of Pathology, King Saud Hospital, Unaizah, Saudi Arabia,; 5 Department of Radiology, Dar Alshefa Hospital, Ibb, Yemen

**Keywords:** Testicular necrosis, epididymo-orchitis, orchiectomy, complication, case report

## Abstract

The symptoms of epididymo-orchitis (EO) are usually mild, but serious complications such as abscess and testicular necrosis can occur. There are a few cases of testicular necrosis secondary to EO to our knowledge. We present a case of a 60-year-old diabetic male patient who presented with left scrotal pain and fever in the last week. The scrotal ultrasonography (US) revealed increased flow of the left testicle in favour of the left EO. After seven days of antibiotic therapy, the patient´s condition worsened and developed into a scrotal abscess. The scrotal US showed scrotal abscess with the absence of left testicular arterial vascularity in favour of testicular necrosis. For that, a left orchiectomy was performed, and a histopathology report confirmed the diagnosis. In conclusion, testicular necrosis secondary to EO is a rare occurrence. When there is a suspicion of EO, medical therapy should be started as soon as possible to avoid significant complications.

## Introduction

Epididymo-orchitis (EO) is a common cause of scrotal pain, accounting for approximately 600,000 cases in the United States each year [1]. Clinical features and physical exams are usually used to diagnose. However, ultrasound (US) can help diagnose and progress this infection [2]. If there is any suspicion of EO, medical treatment should be started as soon as possible to avoid critical complications such as abscessing testis tissue or testicular infarction due to congestion of the spermatic cord and decreased blood flow [3]. Testicular necrosis is a rare complication of EO, occurring in 1-2% of all cases, without a solid guideline to manage this complication [4]. There are few reports of patients with testis necrosis following EO [4,5]. Therefore, we present a case of acute left EO in a 60-year-old diabetic man complicated by abscess formation that results in testis necrosis.

## Patient and observation

**Figure 1:**
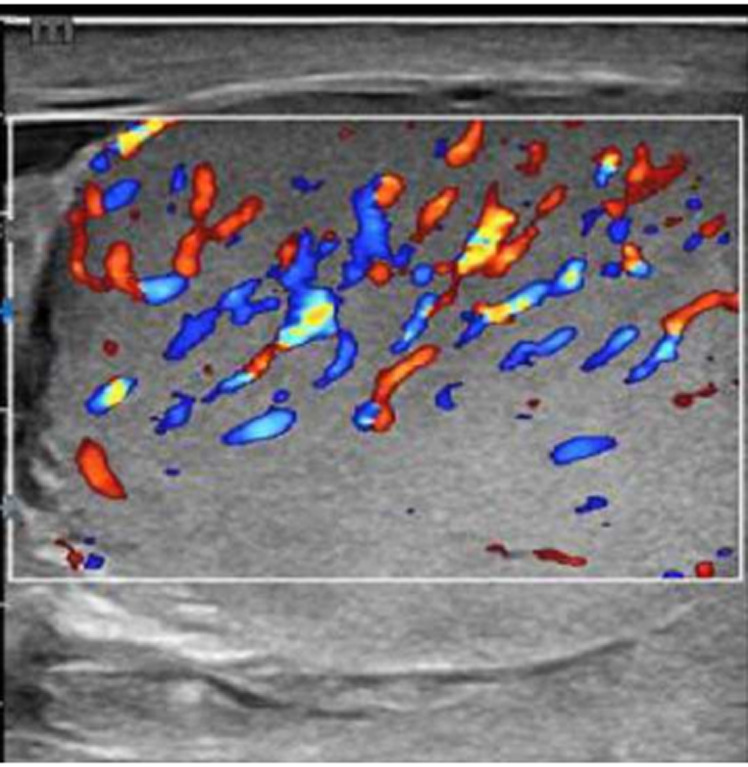
ultrasonography of the left testicle showing increased arterial vascularity in favour of epididymo-orchitis

**Patient information:** a 60-year-old man, a known case of diabetes mellitus on oral hypoglycemic medications, was presented to our urology department in September 2021 with a one-week history of progressive left scrotal pain and high-grade fever. The pain was not radiated, worse in movement, and relieved with analgesic therapy. The patient had a history of dysuria and frequency with few purulent urethral discharges. The patient had no history of trauma, unprotected sexual activity, or similar episode in the past. Otherwise, his medical history was unremarkable. Doppler US revealed increased left testicular flow and high epididymal vascularity that were basically considered EO (Figure 1). Intravenous cefepime 1 g every 12 hours, paracetamol 1 g 8 hours, oral ibuprofen 400 mg 8 hours, and scrotal elevation were recommended for him one week ago. Now, he developed severe left scrotal pain and scrotal discharge.

**Figure 2:**
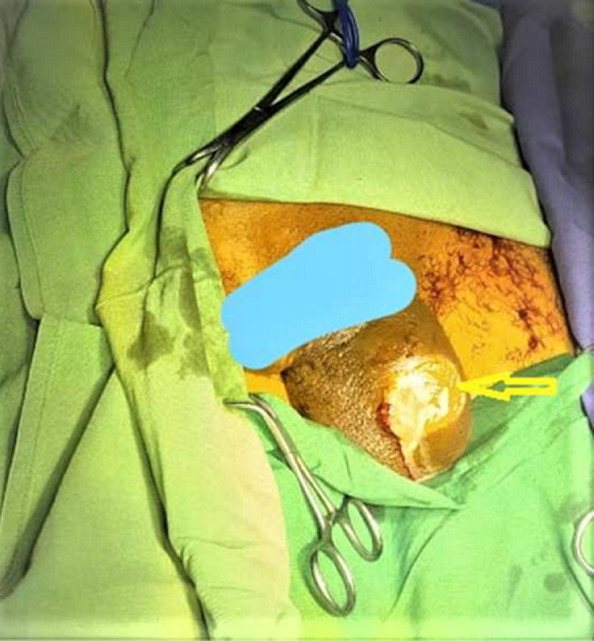
abscess formation (arrow)

**Clinical findings:** the patient´s vital sign was stable on physical examination, and a high-grade fever was detected (oral temperature: 38.5°C). Ecchymosis and edema of the scrotum associated with few purulent discharges from the scrotal wall were observed in urologic genitals examination, suggestive of scrotal abscess formation (Figure 2).

**Figure 3:**
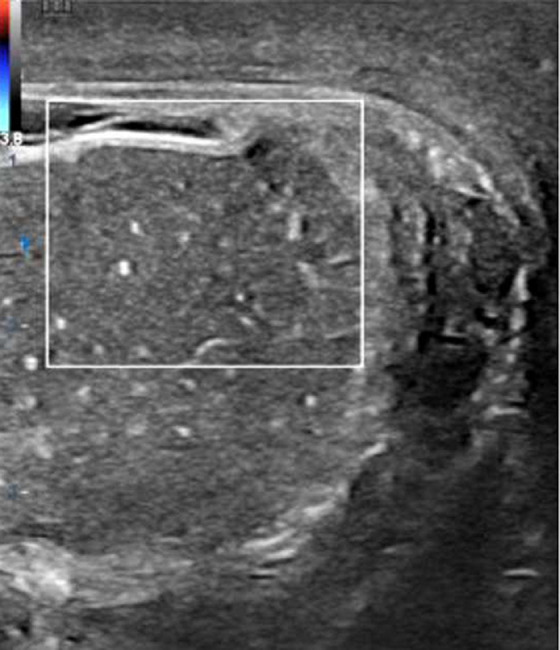
ultrasonography of the left testicle showing the absence of arterial vascularity

**Diagnostic assessment:** urine analysis showed microscopic hematuria and pyuria (5-10 RBC / HPF and 15-20 WBC / HPF of pus cells). Blood investigation revealed white blood cells: 25 x 10^3^/ml with an increase in absolute neutrophil, hemoglobin: 12.4 g/dl, serum creatinine: 1.1 mg/dl, and blood urea nitrogen (BUN): 45 mg/dl. The viral marker of HBsAg was positive, indicating liver hepatitis B. The results of all other blood tests were within the normal range. The abdominal US was unremarkable and only relived mild enlargement of the prostate. Scrotal Doppler US demonstrated enlargement of the left testis associated with heterogeneous hyperechoic echotexture and an absence of blood flow of the left testis. Additionally, the left epididymis was engorged and associated with a small reactive hydrocele (Figure 3).

**Figure 4:**
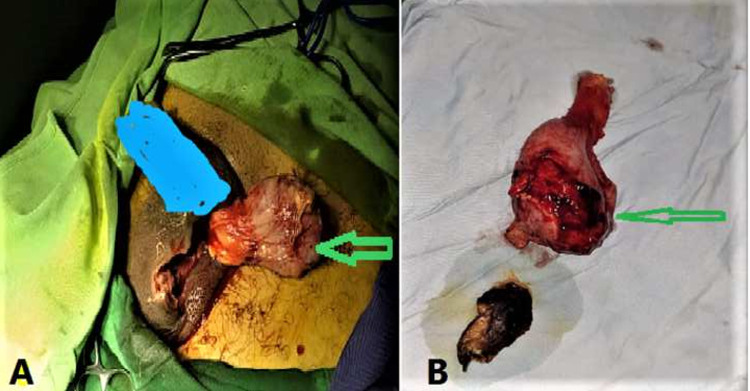
intraoperatively of the left testicle showing: A) necrosis of the left testes (arrow); B) necrotic testes after orchiectomy (arrow)

**Therapeutic interventions:** the patient was admitted for an emergency scrotal exploration. Intraoperative findings relived left testis necrosis and abscess formation. After confirmation of the nonviable testis, a left orchiectomy was performed. Then, the site of the operation was washed with normal slain, the drain was inserted, and the scrotal wall was sutured with nylon 2/0 (Figure 4).

**Figure 5:**
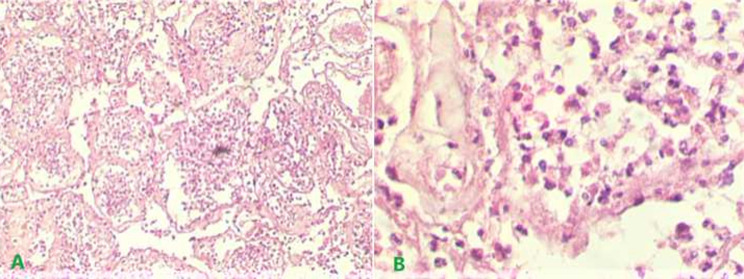
histopathological showing: A) infarcted seminiferous tubules; B) polymorphonuclear infiltration

**Follow-up and outcome:** postoperatively, the patient received intravenous antibiotic (cefepime 1 g every 12 hours) and pain management with intravenous paracetamol 1 g every 8 hours. The drain was removed on the third postoperative day, and the patient was discharged with an oral antibiotic. The histopathological report of left intratesticular abscess formation and testicular necrosis (Figure 5). Within five months of follow-up, no signs of recurrence were recorded.

**Patient perspective:** during treatment, the patient was satisfied with the level of care provided to him.

**Informed consent:** written informed consent was obtained from the patient for participation in our study.

## Discussion

The complications of EO include suppurative necrosis, testis atrophy, infarction, and scrotal abscess formation [6]. Testicular necrosis is a rare complication of EO, with an estimated 1-2% incidence rate [6]. Several reports of testis necrosis after EO, including Elifranji *et al*., Zehri *et al*., Devlies and associations, and Rhudd and associations [2,6-8]. The pathophysiology of testis necrosis caused by EO is unknown. When it happens, a variety of factors are thought to play a role, such as inflammatory infiltration that causes spermatic cord compression, thrombosis due to venous congestion, and bacterial endotoxins [9]. Color Doppler US has become the imaging modality, especially for ruling out testicular torsion, necrosis, and evaluating scrotal contents. The sensitivity and specificity of US for detecting testis torsion were 100% and 75.2%, respectively [10]. Our case reflects the above research results, with our US findings indicating EO within the first presentation and recognizing necrosis of the testis and abscess formation during the second US. *N. gonorrhoeae* and *C. trachomatis* are the most common infectious causes of EO in younger patients, while *E. coli, enterococci, pseudomonas*, and *proteus* are the most common causes of EO in more than 35 [11].

EO´s antibiotic therapy is typically administered with a third-generation Cephalosporin and a Quinolone. However, there is a lack of meaningful evidence to support the clinical characteristics that predict a poor outcome of antibiotic therapy, such as septicemia, noticeable scrotal edema and inflammation, and severe testes pain [4]. If the urine culture result was positive, complications such as hydrocele, abscess formation, testicular infarction, and future infertility could increase. Typically, this confirmation is too late to guide a specific surgical intervention [2,7,8]. Our case had severe scrotal pain and fever, which may indicate a poor outcome of antibiotic therapy. The urine culture of our patient was performed in another laboratory, and we did not have access to the result. Due to testis´ color discoloration and loss of parenchymal tissue complicated by abscess, orchiectomy was unavoidable in our case. Several authors mentioned a similar decision to orchiectomy, such as Alharbi *et al*. who performed orchiectomy in an 18-year-old man with EO complicated by testicular ischemia [4]. Anticoagulation, antiplatelet, and thrombolytic drugs are alternative theoretical management options. Another option is testis and spermatic cord fasciotomy to save the testicle [7,12].

Other authors have attempted to decompress the epididymis and spermatic cord in specific instances, with varying degrees of success [13]. Testicular fasciotomy may appear to be an overly aggressive alternative to orchiectomy. Nevertheless, performing an orchiectomy in a patient with a single testis or subfertility may reduce fertility reproduction [6,14]. As reported, firm evidence to explain testicular necrosis after EO has not been obtained yet. The scarcity of this concept, the impossibility of establishing the diagnosis at the point of diastolic flow reversal, and the lack of guidelines will restrict the standardized approach and optimal management [2]. As validated by histopathological study and US images, our case was a case of acute EO with no response to antibiotic therapy, complicated by abscesses and necrosis of the left testicle. Our case was similar to previously published cases [4,5].

## Conclusion

EO is a common urologic condition that can lead to severe complications such as testicular necrosis. If EO is suspected, we recommend that medical treatment be initiated as soon as possible. Additionally, scrotal Doppler US should be used to diagnose and monitor EO.
